# Simplifying cardiovascular magnetic resonance pulse sequence terminology

**DOI:** 10.1186/s12968-014-0103-z

**Published:** 2014-12-31

**Authors:** Matthias G Friedrich, Chiara Bucciarelli-Ducci, James A White, Sven Plein, James C Moon, Ana G Almeida, Christopher M Kramer, Stefan Neubauer, Dudley J Pennell, Steffen E Petersen, Raymond Y Kwong, Victor A Ferrari, Jeanette Schulz-Menger, Hajime Sakuma, Erik B Schelbert, Éric Larose, Ingo Eitel, Iacopo Carbone, Andrew J Taylor, Alistair Young, Albert de Roos, Eike Nagel

**Affiliations:** Philippa and Marvin Carsley Cardiovascular MR Centre at the Montreal Heart Institute, Université de Montreal, Montreal, Canada; Bristol Heart Institute, University of Bristol, Bristol, UK; Stephenson CMR Centre at the Libin Cardiovascular Institute of Alberta, Calgary, Canada; University of Leeds, Leeds, UK; The Heart Hospital, London, UK; Hospital Santa Maria, Lisbon University, Lisbon, Portugal; Cardiovascular Imaging Center, University of Virginia Health System, Charlottesville, VA USA; Centre for Clinical MR Research, John Radcliffe Hospital, University of Oxford, Oxford, UK; Royal Brompton Hospital, National Heart and Lung Institute, Imperial College, London, UK; NIHR Cardiovascular BRU at Barts, William Harvey Research Institute, Queen Mary University of London, London, UK; Brigham and Women’s Hospital, Harvard Medical School, Boston, MA USA; University of Pennsylvania Medical Center, Philadelphia, PA USA; Charité Universitätsmedizin Berlin and HELIOS-Klinikum, Berlin, Buch Germany; Mie University Hospital, Mie, Japan; UPMC Heart & Vascular Institute, University of Pittsburgh, PA, USA; Institut Universitaire de Cardiologie et de Pneumologie de Québec, Université Laval, Québec City, QC, Canada; Klinik für Innere Medizin/Kardiologie, Herzzentrum/Universität Leipzig, Leipzig, Germany; Università “La Sapienza”, Roma, Italy; Alfred Hospital and Baker Heart and Diabetes Institute, Melbourne, Australia; Auckland University, Auckland, New Zealand; Department of Radiology, Leiden University Medical Center, Leiden, The Netherlands; King’s College, London, UK; Departments of Cardiology and Radiology, Montreal Heart Institute/Université de Montréal, 5000 Rue Belanger, Montréal, QC H1T 1C8 Canada; Departments of Cardiac Sciences and Radiology, Montreal Heart Institute, University of Calgary, 5000 Rue Belanger, Montréal, QC H1T 1C8 Canada

## Abstract

We propose a set of simplified terms to describe applied Cardiovascular Magnetic Resonance (CMR) pulse sequence techniques in clinical reports, scientific articles and societal guidelines or recommendations. Rather than using various technical details in clinical reports, the description of the technical approach should be based on the purpose of the pulse sequence. In scientific papers or other technical work, this should be followed by a more detailed description of the pulse sequence and settings. The use of a unified set of widely understood terms would facilitate the communication between referring physicians and CMR readers by increasing the clarity of CMR reports and thus improve overall patient care. Applied in research articles, its use would facilitate non-expert readers’ understanding of the methodology used and its clinical meaning.

## Background

CMR is considered the non-invasive gold standard for many quantitative measurements in cardiovascular disease. It has been repeatedly shown that CMR is a useful diagnostic tool for a large variety of indications such as cardiomyopathy, myocarditis, right ventricular disease, congenital heart disease, myocardial iron assessment, myocardial ischemia and viability. There are published standards on CMR indications, data acquisition [1], and recommendations on how to interpret [2] and to report [3,4] CMR scans. While frequently used in patient management in tertiary care institutions, CMR is less well established in community hospitals and private practices. Many referring physicians have little or no training in this technique and therefore lack knowledge of CMR principles and terminology. Several reasons exist for this familiarity gap, which may cause difficulties in selecting an appropriate testing strategy for a given clinical problem. First, because of the complex underlying physics, the technological terms often include descriptions of the type, timing, repetitiveness and duration of the pulse sequence technique. Hence, publications and, more importantly, clinical reports often use technical terms that are not intuitively understood by the referring physician or non-CMR-expert and do not convey relevant information or contribute to the quality of the report. Second, these terms often refer to different aspects of the methodology. “First-pass perfusion”, for example describes a time period rather than a sequence, whereas “T2* mapping” relates to the magnetic relaxation time as a physical parameter of the myocardium. Third, multiple and sometimes vendor-specific terms are currently used for the same phenomenon, such as “delayed hyperenhancement” and “late gadolinium enhancement”.

Simplifying CMR pulse sequence terminology could improve the acceptance and widespread application of CMR in clinical routine. We therefore propose a simplified terminology for describing CMR techniques when reporting CMR results in clinical and academic practice, medical publications, as well as in guidelines or other societal recommendations.

### Approach

Given that the primary goal of clinical CMR reports is to provide a concise description of the clinically relevant findings, rather than details of technical aspects of the study, we suggest simplifying CMR sequence terminology. MR-specific technical terms such as generic sequence names should be available as a technical glossary upon request.

Clinical research papers should present such information as part of the methods section, but not in abstracts. Publications on MR technology are not subject to these recommendations.

We are aware that a simplification of the terminology comes at the cost of accuracy with respect to details of the applied protocols. While exact knowledge of the sequence details may be occasionally relevant for follow-up scans, such detailed information however is not contained in the sequence name anyway. Instead, the overall impact of using less complex terms on the application and its benefit to patients outweigh theoretical disadvantages and that the use of arcane, technical language in clinical reports may rather lead to a disconnect between imagers and referring clinicians than to improved trust.

### Proposed terminology in clinical CMR reports

Delivering useful information to referring physicians is the primary objective of clinical reports. The question put forward to the imager should be answered as completely and as specifically as possible, and important additional findings should be reported.

We propose to primarily use terms that incorporate the term CMR, the information on whether the methodology was used for mere visualization or also included quantitative assessment and, wherever possible, the diagnostic target of the pulse sequence.

While previously published recommendations of the Society for Cardiovascular MR (SCMR) list sequence names [3], other societal recommendations on reporting of CMR images do not specifically include listing technical terms [1] or even recommend the use of more generic terms [4,5]. Of note, the use of the detailed pulse sequence description may be helpful for follow-up scans and thus should be included where appropriate, at the discretion of the reporting physician in the technical section of the report. In the more narrative section of a report however, non-generic technical language should be avoided. The term “Late gadolinium enhancement (LGE) CMR” represents an exception. Since this approach is not specific for a certain tissue pathology but may in fact reflect necrosis, fibrosis, infiltration or other causes for an increased volume of distribution of gadolinium, the term LGE can be used as such, followed by its diagnostic target in this particular scan. Table 1 lists currently used terms for frequently implemented sequences and the proposed simplified terminology to present them. This list applies to all MR systems, regardless of the field strength or other technical variations. If new sequences are developed for new purposes, a new, similarly clear term should be identified and used.

**Table 1:** **Proposed simplified CMR sequence terminology**

**Used terms (examples)**	**Term suggestions for clinical reports**	**Modifiers for scientific/technical publications**
Black-blood T1-weighted (half-Fourier, single-shot, fast spin echo, double-inversion recovery) dark blood spin echo sequence with or without contrast agent; with or without fat saturation, proton-density weighted spin echo sequence	Black-blood CMR	… using [sequence name, details*] (applies to all examples)
2D/3D inversion-recovery gradient echo sequence	Late gadolinium enhancement (LGE) CMR	
Regular/single-shot 2D/3D phase-sensitive SSFP		
Delayed (hyper) enhancement sequence		
T2-weighted single-shot/fast spin echo double-inversion recovery/triple-inversion recovery dark/black blood spin echo sequence/T2-prepared SSFP with/without fat saturation	Edema CMR	
Balanced steady-state- free-precession gradient echo sequence, spoiled gradient echo cine sequence with/without contrast agent	Cine CMR	
Gradient echo cine sequence with spatial modulation of magnetization (SPAMM)	Strain CMR	
Steady-state- free-precession or spoiled gradient echo sequence with tissue motion analysis (e.g. feature tracking)		
T1-weighted saturation recovery gradient echo sequence with echo-planar, SSFP, or hybrid read-out	Perfusion CMR	
In plane/through-plane motion-encoded phase-sensitive spoiled gradient echo sequence	Flow CMR	
4D phase contrast velocity sequence		
T2*-weighted spoiled gradient echo sequence	Iron CMR	
T2* mapping sequence		
Time-Of-Flight MR coronary angiography sequence with/without contrast SSFP MR coronary angiography sequence	CMR Coronary Angiography	

*: “Details” indicates any specific methodology used for data acquisition, post-processing and evaluation.

The conclusion of the clinical report should translate the findings described in the body of the report into clinically meaningful information and, if possible, propose a diagnosis that appears most consistent with the findings. Thus, descriptors of the technique are not required at all.

### Proposed terminology in scientific reports and publications

When describing CMR pulse sequences in experimental, preclinical or clinical reports submitted to journals and other media, a detailed and accurate description of hardware and sequences is essential. Therefore, a simplification of the descriptive terms should be avoided. Yet, a detailed technical description can be preceded by terms which can be understood by readers outside the field of CMR. Thus, we propose to use the more commonly understood terms (such as flow CMR) followed by the detailed sequence name.

A list of terms should be part of societal (e.g. Society for Cardiovascular Magnetic Resonance (SCMR)) recommendations, based on a careful evaluation of the existing body of evidence, and distributed to journals and media. The set of recommendations should respond to applications from the SCMR community with periodic updates of the recommended term list.

We strongly encourage clinical CMR readers, scientists, MR equipment and software vendors to consider these recommendations as a service to the community and their patients.

### Summary

We propose the use of more commonly understood terms for the description of CMR protocols in clinical CMR reports that should include the purpose of the sequence and the modality (CMR). In technical or scientific publications, this should be followed by the detailed name of the pulse sequence and any specific approach used for post-processing and evaluation.
